# Underrepresentation of Phenotypic Variability of 16p13.11 Microduplication Syndrome Assessed With an Online Self-Phenotyping Tool (Phenotypr): Cohort Study

**DOI:** 10.2196/21023

**Published:** 2021-03-16

**Authors:** Jianqiao Li, Margaret A Hojlo, Sampath Chennuri, Nitin Gujral, Heather L Paterson, Kent A Shefchek, Casie A Genetti, Emily L Cohn, Kara C Sewalk, Emily A Garvey, Elizabeth D Buttermore, Nickesha C Anderson, Alan H Beggs, Pankaj B Agrawal, John S Brownstein, Melissa A Haendel, Ingrid A Holm, Joseph Gonzalez-Heydrich, Catherine A Brownstein

**Affiliations:** 1 Division of Genetics and Genomics Boston Children's Hospital Boston, MA United States; 2 The Manton Center for Orphan Disease Research Boston Children's Hospital Boston, MA United States; 3 Department of Psychiatry and Behavioral Sciences Boston Children's Hospital Boston, MA United States; 4 Tommy Fuss Center for Neuropsychiatric Disease Research Boston Children's Hospital Boston, MA United States; 5 Innovation and Digital Health Accelerator Boston Children’s Hospital Boston, MA United States; 6 Department of Environmental and Molecular Toxicology Oregon State University Corvallis, OR United States; 7 Computational Epidemiology Group Boston Children’s Hospital Boston, MA United States; 8 Human Neuron Core, Translational Neuroscience Center Boston Children’s Hospital Boston, MA United States; 9 Department of Neurology Boston Children’s Hospital Boston, MA United States; 10 Department of Pediatrics Harvard Medical School Boston, MA United States; 11 Division of Newborn Medicine Boston Children’s Hospital and Harvard Medical School Boston, MA United States; 12 Center for Health Artificial Intelligence University of Colorado Anschutz Aurora, CO United States; 13 Department of Psychiatry Harvard Medical School Boston, MA United States

**Keywords:** self-phenotyping, 16p13.11 microduplication syndrome, copy number variation, genetics, incomplete penetrance, phenotype, variable presentation, human phenotype ontology, online survey, digital health

## Abstract

**Background:**

16p13.11 microduplication syndrome has a variable presentation and is characterized primarily by neurodevelopmental and physical phenotypes resulting from copy number variation at chromosome 16p13.11. Given its variability, there may be features that have not yet been reported. The goal of this study was to use a patient “self-phenotyping” survey to collect data directly from patients to further characterize the phenotypes of 16p13.11 microduplication syndrome.

**Objective:**

This study aimed to (1) discover self-identified phenotypes in 16p13.11 microduplication syndrome that have been underrepresented in the scientific literature and (2) demonstrate that self-phenotyping tools are valuable sources of data for the medical and scientific communities.

**Methods:**

As part of a large study to compare and evaluate patient self-phenotyping surveys, an online survey tool, Phenotypr, was developed for patients with rare disorders to self-report phenotypes. Participants with 16p13.11 microduplication syndrome were recruited through the Boston Children's Hospital 16p13.11 Registry. Either the caregiver, parent, or legal guardian of an affected child or the affected person (if aged 18 years or above) completed the survey. Results were securely transferred to a Research Electronic Data Capture database and aggregated for analysis.

**Results:**

A total of 19 participants enrolled in the study. Notably, among the 19 participants, aggression and anxiety were mentioned by 3 (16%) and 4 (21%) participants, respectively, which is an increase over the numbers in previously published literature. Additionally, among the 19 participants, 3 (16%) had asthma and 2 (11%) had other immunological disorders, both of which have not been previously described in the syndrome.

**Conclusions:**

Several phenotypes might be underrepresented in the previous 16p13.11 microduplication literature, and new possible phenotypes have been identified. Whenever possible, patients should continue to be referenced as a source of complete phenotyping data on their condition. Self-phenotyping may lead to a better understanding of the prevalence of phenotypes in genetic disorders and may identify previously unreported phenotypes.

## Introduction

16p13.11 microduplication syndrome is a rare chromosome duplication syndrome associated with copy number variation (CNV) at the chromosome 16p13.11 locus. The syndrome has high variability in phenotype. Clinical case reports have shown that patients with 16p13.11 microduplication (dup16p13.11) may experience intellectual disability, speech delay, and emotional and behavioral disorders like attention-deficit/hyperactivity disorder (ADHD) and autism spectrum disorder (ASD) [1-4]. Other neurodevelopmental phenotypes related to this CNV include perinatal hypotonia and feeding difficulties, gross motor delay, epilepsy, and schizophrenia [1,3,5]. Abnormal brain magnetic resonance imaging findings have also been described [1-4]. Additional medical conditions related to 16p13.11 microduplication include cardiovascular disease, as well as a range of congenital abnormalities of varying severity including pulmonary stenosis, coarctation of the aorta, thoracic aortic aneurysm dissection, hypermobile joints, hand/foot deformities, microcephaly and macrocephaly, umbilical hernia, and vision problems such as strabismus, myopia, and amblyopia [4,5].

There are considerable challenges in predicting the clinical outcomes of those with 16p13.11 microduplication. For one, there is incomplete penetrance in which both affected and unaffected members from the same family have been found to carry the same CNV, while *de novo* cases also occasionally occur [2-5]. In addition, variable expressivity of the dup16p13.11 phenotypes may occur as a result of the size of the duplication, reportedly ranging from several kilobases to a few megabases. A majority of the known 16p13.11 microduplications include duplication of the gene *NDE1*, which has long been suggested as the primary candidate gene for the neurological and behavioral phenotypes in affected patients [1,4,6-8].

A potentially powerful approach to understanding the complex phenotypic spectrum of 16p13.11 microduplication syndrome is to collect phenotypic data from patients themselves (or their caregivers), as they experience the symptoms and effects of their condition. GenomeConnect, the National Institutes of Health–funded Clinical Genome Resource (ClinGen) patient registry, developed a patient self-phenotyping survey, which asks patient-friendly questions that have been mapped to a set of high-level human phenotype ontology (HPO) terms [9-11]. HPO is a standardized vocabulary of phenotypic abnormalities encountered in human disease, whereby symptoms and characteristic phenotypic findings (a phenotypic profile) are captured using a logically constructed hierarchy of phenotypic terms [12,13]. An alternative method for self-phenotyping is for patients to generate HPO terms for their condition directly. Our group developed a “layperson” HPO survey called “Phenotypr“ to capture patient phenotypes by translating most standard HPO terms into layperson language that would be easy for patients to comprehend and use (eg, a layperson term for “hypotonia” would be “muscle weakness”) [14,15]. We tested the GenomeConnect and Phenotypr surveys computationally and in patients with known rare diseases.

Here, we describe the results of a subset of participants in the larger study who had 16p13.11 microduplication syndrome and completed the Phenotypr survey. The primary aim was to determine if there were self-identified phenotypes that were underrepresented in previous reports of patients with 16p13.11 microduplication syndrome.

## Methods

### Recruitment

Participants were the caregivers, parents, or legal guardians of individuals with 16p13.11 microduplication or the affected individuals themselves, and were aged 18 years or older. Individuals with 16p13.11 microduplication were recruited through the Boston Children’s Hospital online 16p13.11 Participant Registry. Individuals who had previously joined the Registry and self-reported a 16p13.11 microduplication diagnosis were sent an informational email about the Phenotypr study. In addition, a blurb about the study that included research team contact information was posted on 16p13.11 microduplication Facebook groups, and potential participants contacted us directly. Participants received a US $15 Amazon gift card for completing the Phenotypr survey. Participants provided chromosomal microarray reports to confirm diagnoses. The study was approved by the Boston Children’s Hospital Institutional Review Board.

### Phenotypr Survey Development

**Figure 1:**
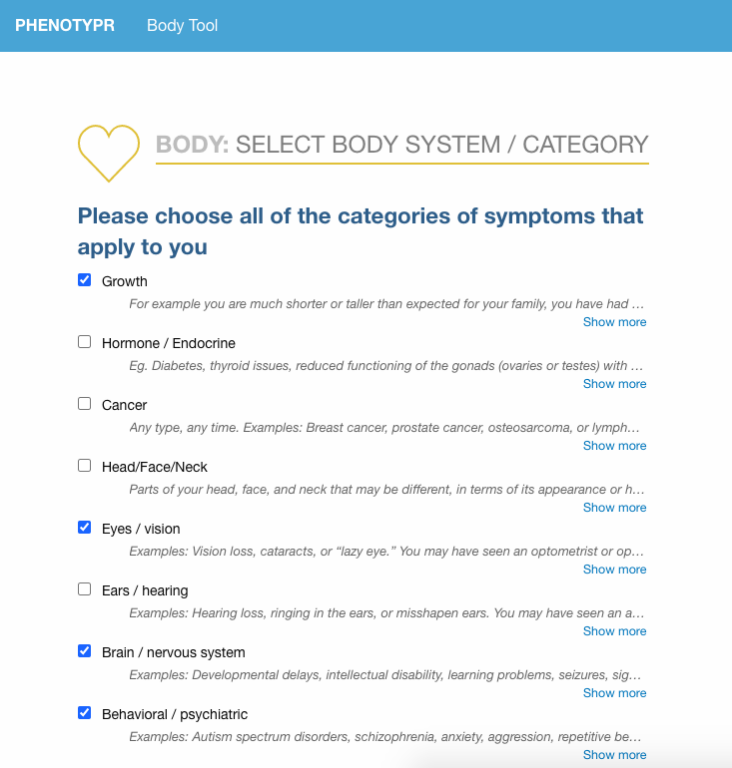
Data entry for Phenotypr: relevant body system selection.

**Figure 2:**
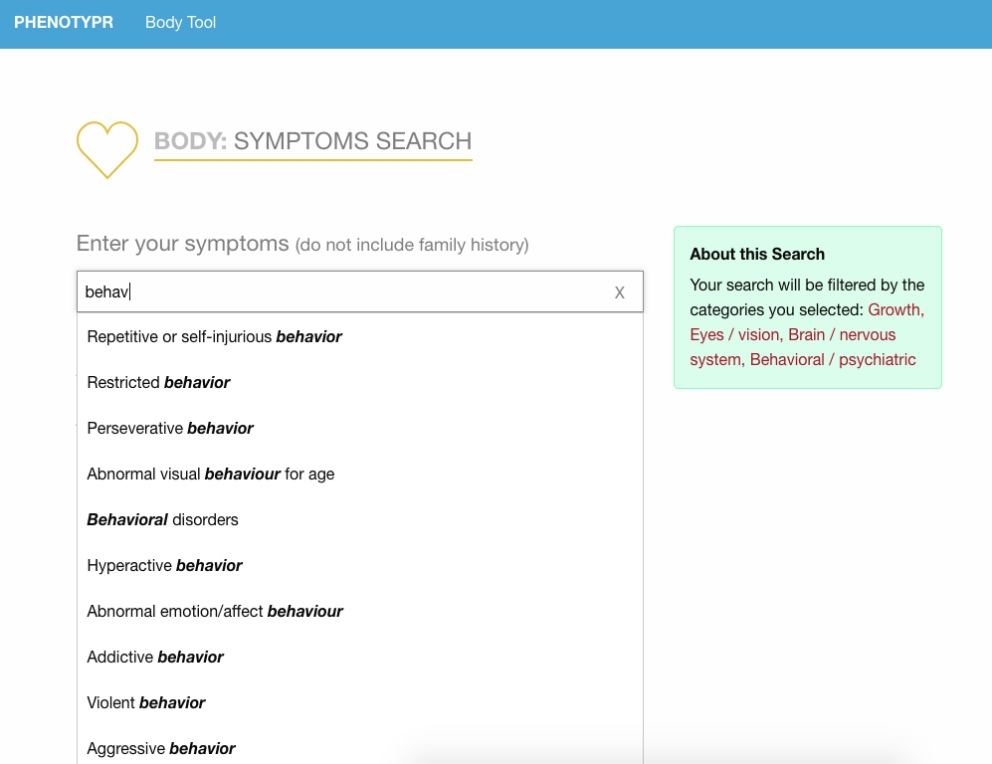
Data entry for Phenotypr: symptom search tool.

**Figure 3:**
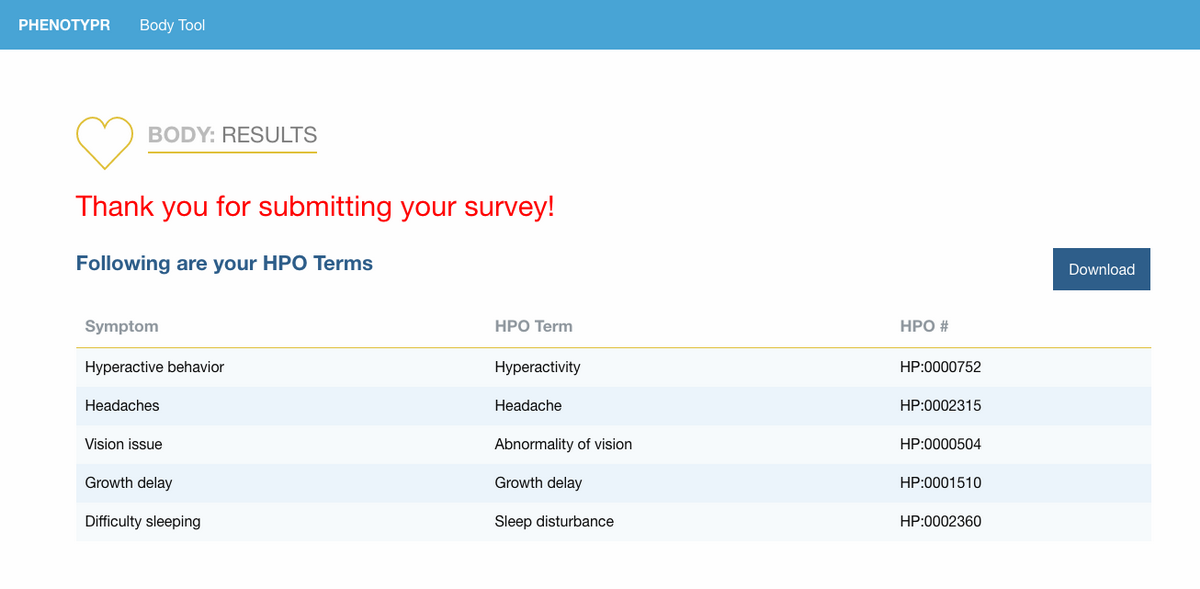
Example of downloadable Phenotypr output.

We developed Phenotypr as a freely available tool that allows patients to record their conditions in layperson and medical HPO terms (see examples of the completion process in Figures 1-3). In Phenotypr, patients first selected the body systems affected by their condition. Seventeen body system options were provided, such as “Growth,” “Ears/Hearing,” and “Brain/Nervous System.” Participants then typed out their symptoms into the symptom search tool, with search result filters applied based on the body systems that they had selected. Tips for entering symptoms were provided, such as reminders to be as specific as possible and include conditions not local to a certain body part (eg, sensitivity to pain). Phenotypr autocompleted each entry with the layperson HPO term, as well as the standard HPO term, and partitioned the terms into anatomically specific sections. The survey ended with a brief demographics form and open-text feedback boxes. Once the survey was complete, the list of the standard HPO terms that corresponded to the layperson HPO terms was downloadable in PDF format.

Phenotypr consisted of a back-end administrative tool for updating ontology versions, user and administrative permissions, and support for alternative implementations; a front-facing public site; and a back-end relational Research Electronic Data Capture (REDCap) database for securely housing the data [16]. Ontology autocomplete features were implemented by processing the HPO and loading structured data into an Apache Solr search engine [17]. The user interface was implemented as a single page application with Vue.js [18].

### Data Collection

Study administrators added participants along with their unique identification number and email address into a secure REDCap database. Participants were then sent an email that contained an invitation and unique link to fill out Phenotypr. The Phenotypr survey was administered via an external web interface and took participants approximately 10 to 15 minutes to complete (Figures 1-3). Each user was assigned a unique token that was carried through to make sure the data were tied to that user. Survey data entered into Phenotypr by participants were temporarily saved in a local Boston Children’s Hospital database.

Two scripts (jobs) were run once every day. The first script imported the newly entered participant information from Boston Children’s Hospital’s internal instance of REDCap and synced it into the local database. The second script exported the survey data (that was filled in by the participant) from the local database into the REDCap instance. The REDCap database included all of the questions that were asked on the external web form. Thus, REDCap provided a secure way to analyze data, create participants, send reminder emails, and manage users. 

Deidentified data were exported into an Excel spreadsheet for further analysis and sharing.

## Results

Nineteen participants enrolled in the study and completed the survey. The caregiver, parent, or legal guardian filled out the Phenotypr survey in 15 of 19 cases. In the other four cases, it was not reported whether the respondent filled out the survey on behalf of themselves or as a caregiver, parent, or legal guardian (Table 1). Table 1 lists the phenotypic characteristics of our cohort as reported in Phenotypr (see Multimedia Appendix 1 for a complete table of phenotypic features).

In order to assess the initial accuracy of the Phenotypr tool, a comparison was conducted between the 16p13.11 microduplication syndrome phenotypes that were present in our cohort and those in a recent case report with a larger sample size [4] (Table 2). Moreover, we compared our cohort to all previously published cases of dup16p13.11 CNV and discovered several underrepresented phenotypes, which are summarized in Table 3 [1,3-5,19].

At least one of the phenotypes mentioned in previous publications, such as delayed speech, learning/intellectual disability, ASD, sleep disorder, and feeding difficulties [1-5], was reported by a majority of the Phenotypr participants (10/19 [53%] cases with at least one phenotype; Table 1). Meanwhile, aggression, which has not been widely reported in previous 16p13.11 microduplication literature, was reported by three families (Table 3).

Additionally, 4 out of 19 (21%) cases mentioned anxiety or anxiety-related behaviors, which is higher than the prevalence of anxiety reported by the Centers for Disease Control and Prevention (approximately 7% in US children aged 3-17 years) [20].

Finally, 5 out of 19 (26%) participants reported immune- and/or autoimmune-related disorders, including severe T-cell immunodeficiency in one patient and “autoimmune encephalopathy and corresponding antibody positivity” in another. Three cases reported asthma, a broadly recognized autoimmune disease, with one individual also having co-occurring hypothyroidism [21].

**Table 1:** Selected phenotypic features in 19 cases of 16p13.11 microduplication syndrome.

Characteristic		Presented cases (patient numbers)																		
		1	2	3	4	5	6	7	8	9	10	11	12	13	14	15	16	17	18	19
Age (years)		10	3	8	2	14	12	14	5	<1	7	6	N/A^a^	N/A	<1	18	N/A	3	9	N/A
**Growth**																				
	Growth abnormality	−^b^	−	+^c^	−	−	−	−	−	−	+	−	−	−	−	−	−	−	−	−
	Muscle weakness	−	−	−	−	−	+	−	−	−	−	−	−	−	−	−	−	−	−	−
	Tall stature	−	−	−	−	−	−	+	−	−	−	−	−	−	−	−	−	−	−	−
**Development**																				
	Delayed speech	−	+	−	−	+	−	−	−	−	+	−	−	−	−	−	−	−	−	−
	Developmental regression	−	+	−	+	−	−	−	−	−	−	−	−	−	−	−	−	−	−	−
	Intellectual disability	−	−	−	−	+	−	−	−	+	−	−	−	−	−	−	−	−	−	−
	Mild global DD^d^	−	−	−	−	−	−	−	−	−	+	−	−	−	−	−	−	−	−	−
**Neurological and mental**																				
	Specific learning disability	−	−	−	−	+	−	−	+	−	−	−	−	−	−	−	−	−	−	−
	Dyslexia	−	−	−	−	−	−	−	−	−	−	−	+	−	−	−	−	−	−	−
	Cognitive impairment	−	−	−	−	−	−	−	−	−	+	−	−	−	−	−	−	−	−	−
	Hypotonia	−	−	−	+	−	+	−	−	−	+	−	−	−	−	−	−	−	−	−
	Poor fine motor coordination	−	−	−	−	+	−	−	−	−	+	−	−	−	−	−	−	−	−	−
	Tics	−	−	−	−	−	+	+	−	−	−	−	−	−	−	−	−	−	−	−
	Spasticity	−	−	−	−	−	−	−	−	−	+	−	−	−	−	−	−	−	−	−
	Dysarthria	−	−	−	+	+	−	−	−	−	−	−	−	−	−	−	−	−	−	−
	Anxiety	−	−	−	−	−	−	+	−	−	−	−	−	−	−	+	−	−	−	−
	Depression	−	−	−	−	−	−	+	−	−	−	−	−	−	−	−	−	−	−	−
	ODD^e^	−	−	−	−	−	−	−	+	−	−	−	−	−	−	−	−	−	−	−
	ADHD^f^	−	−	−	−	+	−	−	−	−	−	−	−	−	−	−	−	−	−	−
	Seizure	−	−	−	−	−	−	−	+	−	+	−	−	−	−	−	−	−	−	+
	ASD^g^/autistic behavior	−	−	−	−	+	+	−	−	−	+	+	−	−	−	−	−	−	−	−
	Sleep disturbance	−	−	−	−	+	+	−	−	+	+	−	−	−	−	−	−	−	−	−
	Parasomnia	−	−	−	−	−	−	−	−	−	+	−	−	−	−	−	−	−	−	−
**Behavior**																				
	Behavioral abnormality	−	−	−	−	+	−	−	−	−	−	−	−	−	−	−	−	−	−	+
	Impulsiveness or violence	−	−	−	−	+	−	−	−	−	+	−	−	−	−	−	−	−	−	−
	Aggression	−	−	−	−	+	−	−	+	−	+	−	−	−	−	−	−	−	−	−
	Abnormal eating	−	−	−	−	−	−	−	−	−	+	−	−	−	−	−	−	−	−	−
	Self-mutilation	−	−	−	−	−	−	−	−	−	+	−	−	−	−	−	−	−	−	−
	Abnormal fear/anxiety	−	−	−	−	−	−	−	−	−	+	−	−	+	−	−	−	−	−	−
	DMDD^h^	−	−	−	−	+	−	−	−	−	+	−	−	−	−	−	−	−	−	−
**Sensory**																				
	Sensory impairment	−	−	−	+	−	−	+	−	−	−	−	−	−	−	−	−	−	−	−
	Hearing impairment	−	−	+	−	−	−	+	−	−	+	−	−	−	−	−	−	−	−	−
	Tinnitus	−	−	−	−	−	+	−	−	−	−	−	−	−	−	−	−	−	−	−
	Astigmatism	−	−	−	−	−	−	−	+	−	−	−	−	−	−	−	−	−	−	−
**Immunity**																				
	Abnormality of the immune system	−	−	+	−	−	−	−	−	−	−	−	−	−	−	−	−	−	−	−
	Severe T-cell immunodeficiency	−	−	+	−	−	−	−	−	−	−	−	−	−	−	−	−	−	−	−
	Autoimmune antibody positivity	−	−	−	−	−	+	−	−	−	−	−	−	−	−	−	−	−	−	−
	Autoimmune encephalopathy	−	−	−	−	−	+	−	−	−	−	−	−	−	−	−	−	−	−	−
**Cardiac and respiratory**																				
	Arrhythmia	−	−	−	−	−	−	−	−	−	−	−	−	−	−	+	−	−	−	−
	Bradycardia	−	−	+	−	−	−	−	−	−	−	−	−	−	−	−	−	−	−	−
	Asthma	−	−	−	−	−	−	−	−	−	−	−	−	−	−	+	−	+	−	+
	Chronic lung disease	−	−	−	+	−	−	−	−	−	−	−	−	−	−	−	−	−	−	−
	Neonatal respiratory distress	−	−	−	−	−	−	−	−	−	+	−	−	−	−	−	−	−	−	−
	Breathing dysregulation	−	−	−	−	−	−	−	−	−	−	−	−	−	+	−	−	−	−	−
**Feeding difficulties**																				
	Gastrostomy tube feeding in infancy	−	−	−	+	−	−	−	−	−	−	−	−	−	−	−	−	−	−	−
	Dysphagia	−	−	−	−	−	−	−	−	−	+	−	−	−	−	−	−	−	−	−
	Feeding difficulties	−	−	−	+	−	−	−	−	−	+	−	−	−	−	−	−	−	−	−

^a^N/A: not applicable.

^b^Feature absent or undisclosed.

^c^Feature present.

^d^DD: developmental delay.

^e^ODD: oppositional defiant disorder.

^f^ADHD: attention-deficit/hyperactivity disorder.

^g^ASD: autism spectrum disorder.

^h^DMDD: disruptive mood dysregulation disorder.

**Table 2:** Comparison of the self-phenotyping cohort with the published cohort in the study by Allach El Khattabi et al [4].

Feature			Cases in the study by Allach El Khattabi et al [4] (N=45)	Presented cases (N=19)
Hypotonia			5/45	3/19
Feeding difficulties			5/45	2/19
**Neurodevelopmental features**				
	Developmental delay^a^		32/45	3/19
	Motor delay^b^		19/45	2/19
	Speech delay		35/45	3/19
	Learning disabilities		30/45	2/19
	ASD^c^		24/45	4/19
	Aggression		—^d^	3/19
	Anxiety		—	4/19
	Seizures		10/45	3/19
	Sleep disorders^e^		8/45	4/19
**Craniofacial features**				
	Microcephaly		1/23	1/19
**Abnormal extremities**				
	**Hands**			
		Long fingers	1/22	1/19
	**Eyes**			
		Strabismus	6/45	1/19
		Myopia	4/45	1/19
		Amblyopia	1/23	1/19
		Nystagmus	1/22	1/19
**Immunological disorders**				
	Immunodeficiency and autoimmune diseases		—	2/19
**Others**				
	Asthma		—	3/19
	Hypothyroidism		—	1/19
	Umbilical hernia		1/22	1/19
	Hearing loss^f^		1/23	3/19
	Abnormality of the male genitalia		2 testicular ectopia and 1 cryptorchidism	1/19

^a^Including “developmental regression” and “mild global developmental delay.”

^b^Corresponding to “poor fine motor coordination.”

^c^ASD: autism spectrum disorder.

^d^Not available.

^e^Including “sleep disturbance” and “parasomnia.”

^f^Corresponding to “hearing impairment.”

**Table 3:** Comparison of the self-phenotyping cohort with published case reports for anxiety, aggression, asthma, and immunological disorder phenotypes.

Phenotype	Cases in the study by Hannes et al [5] (N=5)	Cases in the study by Ramalingam et al [1] (N=8)	Cases in the study by Nagamani et al [3] (N=10)	Cases in the study by Loureiro et al [19] (N=4)	Cases in the study by Allach El Khattabi et al [4] (N=45)	Cases in this study (N=19)
Anxiety	—^a^	1/8^b^	—	1/4^c^	—	4/19
Aggression	1/5	—	1/10	—	—	3/19
Asthma	—	—	—	—	—	3/19
Immunological disorders^d^	—	—	—	—	—	2/19

^a^Not available.

^b^Co-occurring with autism spectrum disorder and attention-deficit/hyperactivity disorder.

^c^Co-occurring with autism spectrum disorder and hyperactivity.

^d^Including “abnormality of the immune system,” “severe T-cell immunodeficiency,” “autoimmune antibody positivity,” and “autoimmune encephalopathy.”

## Discussion

### Principal Findings

This cohort of 19 16p13.11 microduplication cases expands the knowledge of an increasingly concerning syndrome. It supports the role of many clinical features that have been previously described, including growth and behavioral disturbances, seizures, and a spectrum of neurological characteristics (Table 1). Further, the frequency of anxiety and aggression in the cohort illustrates the potential utility of self-phenotyping and nonhypothesis-driven phenotyping tools as informative data sources. The amount of immune disorders reported is also an interesting finding worthy of further investigation. The incidence rate of asthma in the cohort (3/19, 16%) was higher than that in published reports from the Centers for Disease Control and Prevention in 2018 (about 1/13, 8%) [22]. Taken together, these results imply the possibility of a broader impact of the genotype on the immune system.

### Limitations

A limitation of this study is ascertainment bias. The participants were located in geographic areas where they had increased access to clinical microarray technology and formal medical diagnosis, and presented with severe enough phenotypes that the families had sought joining an online registry to participate in research; therefore, it is possible that this cohort is more severely impacted than 16p13.11 microduplication cases in the general population. Patients and families dealing with unique presentations may also be more incentivized to participate in research and use an internet self-phenotyping tool. That said, published studies may also have had similar biases, as they were recruiting participants with otherwise unexplained phenotypes, who went on to have chromosomal microarray testing, thus limiting our assessment on the presence of normal variation. Another limitation comes from the fact that for four out of 19 respondents, the individuals did not indicate whether they were filling out the survey as an individual with 16p13.11 microduplication syndrome or as a caregiver. This can add variability in the responses and cause information bias from surrogate interviews [23].

### Comparison With Prior Work on 16p13.11 Microduplication

New phenotypes associated with the dup16p13.11 CNV have been continuously discovered and reported since the syndrome first came to light in 2007 when Ullmann et al initially identified the 16p13.11 microduplication and believed it predisposed patients to ASD and intellectual disability [2]. Subsequently, further studies have indicated that the 16p13.11 microduplication is likely to be involved with a wide spectrum of neurodevelopmental disorders [1,24-27]. We hereby identified two neuropsychiatric disorders, anxiety and aggression, as supplementary evidence to the underrepresented cases in prior reports, and we underline a probable high occurrence of these two phenotypes in patients with dup16p13.11. As shown in Table 3, only two cases of anxiety have been previously reported; one by Ramalingam et al (1/8 patients) [1] in 2011 and another by Loureiro et al (1/4 patients) [19] in 2017. Although one case was inherited and the other occurred *de novo*, both aforementioned cases were associated with ASD or ADHD, which aligns with literature that anxiety is a common condition in patients with ADHD and ASD [28,29]. In our cases, however, just one individual with anxiety had co-occurring ASD, whereas the remaining three cases of anxiety appeared independently. Meanwhile, aggression was also noted in three family reports, and it has gone largely unreported since Hannes et al first described a case of aggression in a patient with dup16p13.11 in 2009 [5], and another case was reported in the study by Nagamani et al in 2011 [3]. Furthermore, a potential correlation between 16p13.11 microduplication and a range of immunological disorders, specifically autoimmune conditions, was uncovered in multiple individuals in our cohort.

Aggressive behavior is one of the most common reasons for mental health referrals in children and adolescents [30-32], and can co-occur with a broad array of psychiatric and neurological illnesses, including ASD, intellectual disability, ADHD, conduct disorder, oppositional defiant disorder, disruptive mood dysregulation disorder, schizophrenia, epilepsy, anxiety, depression, and sleep disorders [33,34]. While impulsive aggression may not indicate any specific disorder, it is an important marker of severity for many psychiatric diseases [35]. For example, aggression level may affect the decision to initiate or increase medication dosage in pediatric ADHD treatment [36]. Regardless of the controversy over whether to consider impulsive aggression as an independent categorical diagnosis like a disorder [37] or a measurable symptom secondary to some other diagnostic entity like fever or pain [32,38], it can greatly impact an individual’s development and health, or even lead to high economic and medical burden for families and communities [39-41]. This paper does not aim to address the dispute over the clinical perception of aggression by joining any side or to unduly correlate 16p13.11 microduplication with the aggression phenotype. Instead, we hope to draw more attention to the repeatedly mentioned and thus important phenotypes that are self-reported by the patient families and call for more focus on related research and therapeutics.

The potential connection between neuropsychiatric disorders and autoimmunity/immunological dysfunction has received growing interest over the past decades. For instance, a nationwide population-based prospective cohort study in Denmark used a longitudinal registry to find that autoimmune diseases and infection raised the risk for subsequent mood disorder [42]. Another well-covered example is the bidirectional relationship between psychosis and autoimmune disorders [43], specifically schizophrenia and celiac disease, between which positive correlations have been suggested through studies of epidemiology, genetics, and immunology [44,45]. Notably, the discovery of autoimmune encephalitis and the disruptive autoantibody mechanisms behind it provided more direct proof of etiological linkage [46-49]. Autoimmune encephalitis is an abrupt inflammatory brain disease characterized by a variety of neuropsychiatric symptoms, such as cognitive and behavioral alterations, seizures, anxiety, and sleep disturbances [50,51], and responds to immunotherapy treatment in many cases [52,53]. Among all the possible causative autoantibodies, one of the most discussed is the N-methyl-D-aspartate receptor (NMDAR) antibody. The NMDAR antibody targets a certain subunit of NMDAR, a synaptic and neuronal cell membrane protein, and has been revealed to play a role in the development and progression of schizophrenia [47,54-57] and ASD [58-61]. Given the long-lasting belief that ASD is largely genetic [62-66], our finding of several individuals with dup16p13.11 CNV who have immunity-related diseases and neuropsychiatric symptoms, especially a case with both ASD and autoimmune encephalopathy plus corresponding antibody positivity, supports the medical field’s current views and may influence future studies that seek to understand genetic origin.

It is worth noting that new associated phenotypes may emerge even for established genetic diseases as more scientific research is conducted and the demographic profiles of patient populations shift. For example, though the first case of Down syndrome was reported in the 19th century, it was not until the last two decades that individuals with Down syndrome were identified as having an increasing risk of early-onset Alzheimer disease as they age; many individuals with Down syndrome start to develop Alzheimer disease pathology in their 30s and approximately two-thirds have dementia by the age of 60 years [67,68]. Due to advancements in health care and social support, the life expectancy of the Down syndrome population has greatly improved, with the average age of death in developed countries now approaching 60 years [69]. A similar increase in knowledge has occurred regarding Turner syndrome, which was first described in the 1930s and is one of the most common genetic disorders [70,71]. Some patients with Turner syndrome who carried a mosaic 46, XY karyotype or an abnormal Y chromosome were recently found to have a higher risk of developing gonadoblastoma and other gonadal tumors owing to the widespread use and easy availability of polymerase chain reaction technology [7,72]. For genetic diseases with variable presentations and complex genotypes, such as Down syndrome and Turner syndrome, it may be too early to announce that all phenotypes have been exhaustively discovered and included.

We do not claim that Phenotypr is a substitute for clinician phenotyping, and it is unlikely that participants will be able to describe some of the highly technical aspects of their disorders in clinical terminology. Therefore, we do not consider the underreporting of some phenotypes in Phenotypr to be notable. In contrast, phenotypes that were repeatedly mentioned by participants but have not been well discussed in the literature are worthy of further investigation. It is possible that patients and families are not mentioning these phenotypes in clinic visits or that their existing concerns are not being fully understood.

### Conclusions

In this study, we utilized the Phenotypr tool to collect self-phenotyping data from 19 16p13.11 microduplication syndrome cases, with the aim of identifying underrepresented phenotypes in the current scientific literature. A number of phenotypes were highlighted, including aggression, anxiety, and a range of immunological disorders. In addition to the typically recognized phenotypes, dup16p13.11 CNV showed a stronger predisposition to aggression and anxiety compared to previously reported cases, as both phenotypes were mentioned by multiple Phenotypr participant families (4/19, 21%). Moreover, we found that three out of four cases with anxiety did not have co-occurring ASD or ADHD, which differed from two other published cases [1,28]. An interesting case that involved ASD and autoimmune encephalopathy with corresponding antibody positivity was also identified using Phenotypr. These findings illustrate some important hypotheses. First, aggression and anxiety may be more common than previously understood in 16p13.11 microduplication cases. Second, anxiety may appear independently as a result of dup16p13.11 CNV instead of being accompanied with ASD or ADHD. Third, immune and autoimmune disorders might be phenotypes of 16p13.11 microduplication, and dup16p13.11 CNV might play a genetic role in the association between autoimmune encephalopathy and ASD. Fourth, in consideration of the incomplete penetrance and varied expression of this syndrome in a broad spectrum of neuropsychiatric disorders, patients and their clinicians should be aware of all possible phenotypes to ensure that treatment is as effective as possible. Fifth, HPO and layperson HPO profiles acquired through patient self-phenotyping can serve as a valuable data source for the exploration of underreported phenotypes in the scientific literature, especially for rare disorders with variable presentations.

Future work will apply Phenotypr to additional 16p13.11 microduplication cases and correlate phenotypic results to the size of the duplicated interval and the genes involved. We also look forward to adopting Phenotypr as a complementary data source in other genetic cohort studies of rare diseases.

## Appendix

Multimedia Appendix 1 Selected phenotypic features in 19 cases of 16p13.11 microduplication syndrome.

## Abbreviations

ADHD: attention-deficit/hyperactivity disorder
ASD: autism spectrum disorder
CNV: copy number variation
HPO: human phenotype ontology
NMDAR: N-methyl-D-aspartate receptor
REDCap: Research Electronic Data Capture
