# Supplementary material for: Underrepresentation of Phenotypic Variability of 16p13.11 Microduplication Syndrome Assessed With an Online Self-Phenotyping Tool (Phenotypr): Cohort Study
Source: J Med Internet Res. 2021 Mar 16;23(3):e21023. doi: 10.2196/21023 (PMC8074853; doi:10.2196/21023)
Supplement: Multimedia Appendix 1 [file jmir_v23i3e21023_app1.docx]

**Multimedia Appendix 1**. Selected phenotypic features in 19 cases of 16p13.11 microduplication syndrome.

|  |  | Presented cases | | | | | | | | | | | | | | | | | | |
| --- | --- | --- | --- | --- | --- | --- | --- | --- | --- | --- | --- | --- | --- | --- | --- | --- | --- | --- | --- | --- |
|  |  |  |  |  |  |  |  |  |  |  |  |  |  |  |  |  |  |  |  |  |
| Patient No. | | 1 | 2 | 3 | 4 | 5 | 6 | 7 | 8 | 9 | 10 | 11 | 12 | 13 | 14 | 15 | 16 | 17 | 18 | 19 |
| Age (years) | | 10 | 3 | 8 | 2 | 14 | 12 | 14 | 5 | <1 | 7 | 6 | N/A | N/A | <1 | 18 | N/A | 3 | 9 | N/A |
| Growth | |  |  |  |  |  |  |  |  |  |  |  |  |  |  |  |  |  |  |  |
|  | Growth abnormality | - | - | + | - | - | - | - | - | - | + | - | - | - | - | - | - | - | - | - |
|  | Muscle weakness | - | - | - | - | - | + | - | - | - | - | - | - | - | - | - | - | - | - | - |
|  | Tall stature | - | - | - | - | - | - | + | - | - | - | - | - | - | - | - | - | - | - | - |
| Development | |  |  |  |  |  |  |  |  |  |  |  |  |  |  |  |  |  |  |  |
|  | Delayed speech | - | + | - | - | + | - | - | - | - | + | - | - | - | - | - | - | - | - | - |
|  | Developmental regression | - | + | - | + | - | - | - | - | - | - | - | - | - | - | - | - | - | - | - |
|  | Intellectual disability | - | - | - | - | + | - | - | - | + | - | - | - | - | - | - | - | - | - | - |
|  | Mild global DD | - | - | - | - | - | - | - | - | - | + | - | - | - | - | - | - | - | - | - |
|  | Renal hypoplasia | - | - | - | - | - | - | - | - | - | - | - | - | - | + | - | - | - | - | - |
| Neurological and mental | |  |  |  |  |  |  |  |  |  |  |  |  |  |  |  |  |  |  |  |
|  | Specific learning disability | - | - | - | - | + | - | - | + | - | - | - | - | - | - | - | - | - | - | - |
|  | Dyslexia | - | - | - | - | - | - | - | - | - | - | - | + | - | - | - | - | - | - | - |
|  | Cognitive impairment | - | - | - | - | - | - | - | - | - | + | - | - | - | - | - | - | - | - | - |
|  | Hypotonia | - | - | - | + | - | + | - | - | - | + | - | - | - | - | - | - | - | - | - |
|  | Poor fine motor coordination | - | - | - | - | + | - | - | - | - | + | - | - | - | - | - | - | - | - | - |
|  | Tics | - | - | - | - | - | + | + | - | - | - | - | - | - | - | - | - | - | - | - |
|  | Spasticity | - | - | - | - | - | - | - | - | - | + | - | - | - | - | - | - | - | - | - |
|  | Dysarthria | - | - | - | + | + | - | - | - | - | - | - | - | - | - | - | - | - | - | - |
|  | Anxiety | - | - | - | - | - | - | + | - | - | - | - | - | - | - | + | - | - | - | - |
|  | Depression | - | - | - | - | - | - | + | - | - | - | - | - | - | - | - | - | - | - | - |
|  | Impaired social interactions | - | - | - | - | - | - | + | - | - | - | - | - | - | - | - | - | - | - | - |
|  | ODD | - | - | - | - | - | - | - | + | - | - | - | - | - | - | - | - | - | - | - |
|  | ADHD | - | - | - | - | + | - | - | - | - | - | - | - | - | - | - | - | - | - | - |
|  | Seizure | - | - | - | - | - | - | - | + | - | + | - | - | - | - | - | - | - | - | + |
|  | ASD/Autistic behavior | - | - | - | - | + | + | - | - | - | + | + | - | - | - | - | - | - | - | - |
|  | Sleep disturbance | - | - | - | - | + | + | - | - | + | + | - | - | - | - | - | - | - | - | - |
|  | Parasomnia | - | - | - | - | - | - | - | - | - | + | - | - | - | - | - | - | - | - | - |
| Behavior | |  |  |  |  |  |  |  |  |  |  |  |  |  |  |  |  |  |  |  |
|  | Behavioral abnormality | - | - | - | - | + | - | - | - | - | - | - | - | - | - | - | - | - | - | + |
|  | Impulsiveness or violence | - | - | - | - | + | - | - | - | - | + | - | - | - | - | - | - | - | - | - |
|  | Aggression | - | - | - | - | + | - | - | + | - | + | - | - | - | - | - | - | - | - | - |
|  | Abnormal eating | - | - | - | - | - | - | - | - | - | + | - | - | - | - | - | - | - | - | - |
|  | Self-mutilation | - | - | - | - | - | - | - | - | - | + | - | - | - | - | - | - | - | - | - |
|  | Abnormal fear/anxiety | - | - | - | - | - | - | - | - | - | + | - | - | + | - | - | - | - | - | - |
|  | DMDD | - | - | - | - | + | - | - | - | - | + | - | - | - | - | - | - | - | - | - |
| Sensory | |  |  |  |  |  |  |  |  |  |  |  |  |  |  |  |  |  |  |  |
|  | Sensory impairment | - | - | - | + | - | - | + | - | - | - | - | - | - | - | - | - | - | - | - |
|  | Hearing impairment | - | - | + | - | - | - | + | - | - | + | - | - | - | - | - | - | - | - | - |
|  | Tinnitus | - | - | - | - | - | + | - | - | - | - | - | - | - | - | - | - | - | - | - |
|  | Sound sensitivity | - | - | - | - | - | - | - | - | - | + | - | - | - | - | - | - | - | - | - |
|  | Early cutaneous photosensitivity | - | - | - | - | - | - | - | - | - | + | - | - | - | - | - | - | - | - | - |
|  | Astigmatism | - | - | - | - | - | - | - | + | - | - | - | - | - | - | - | - | - | - | - |
|  | Amblyopia | - | - | - | - | - | - | - | - | - | + | - | - | - | - | - | - | - | - | - |
|  | Alternating exotropia | - | - | - | - | - | - | - | - | - | + | - | - | - | - | - | - | - | - | - |
|  | Myopia | - | - | - | - | - | - | - | - | - | - | - | - | - | - | - | - | + | - | - |
| Brain | |  |  |  |  |  |  |  |  |  |  |  |  |  |  |  |  |  |  |  |
|  | EEG abnormality | - | - | - | - | - | + | - | - | - | - | - | - | - | - | - | - | - | - | - |
|  | Cerebral ventricles abnormality | - | - | - | - | + | - | - | - | - | - | - | - | - | - | - | - | - | - | - |
|  | Hypoplasia of the brainstem | - | - | + | - | - | - | - | - | - | - | - | - | - | - | - | - | - | - | - |
| Immunity | |  |  |  |  |  |  |  |  |  |  |  |  |  |  |  |  |  |  |  |
|  | Abnormality of the immune system | - | - | + | - | - | - | - | - | - | - | - | - | - | - | - | - | - | - | - |
|  | Severe T cell immunodeficiency | - | - | + | - | - | - | - | - | - | - | - | - | - | - | - | - | - | - | - |
|  | Autoimmune antibody positivity | - | - | - | - | - | + | - | - | - | - | - | - | - | - | - | - | - | - | - |
|  | Autoimmune encephalopathy | - | - | - | - | - | + | - | - | - | - | - | - | - | - | - | - | - | - | - |
| Gastrointestinal tract | |  |  |  |  |  |  |  |  |  |  |  |  |  |  |  |  |  |  |  |
|  | Gastroparesis | - | - | + | - | - | - | - | - | - | - | - | - | - | - | - | - | - | - | - |
|  | Chronic constipation | - | - | - | - | + | - | - | - | - | + | - | - | - | - | - | - | - | - | - |
|  | Gastroesophageal reflux | - | - | - | - | - | + | - | - | - | + | - | - | - | + | - | - | - | - | - |
|  | Abnormal gastrointestinal motility | - | - | - | - | - | - | - | - | - | + | - | - | - | - | - | - | - | - | - |
|  | Bowel incontinence | - | - | - | - | - | - | - | - | - | - | - | - | - | - | - | - | - | - | + |
| Cardiac and respiratory | |  |  |  |  |  |  |  |  |  |  |  |  |  |  |  |  |  |  |  |
|  | Scimitar anomaly | - | - | - | + | - | - | - | - | - | - | - | - | - | - | - | - | - | - | - |
|  | Prolonged QT interval | - | - | - | - | - | - | - | - | - | + | - | - | - | - | - | - | - | - | - |
|  | Arrhythmia | - | - | - | - | - | - | - | - | - | - | - | - | - | - | + | - | - | - | - |
|  | Bradycardia | - | - | + | - | - | - | - | - | - | - | - | - | - | - | - | - | - | - | - |
|  | Asthma | - | - | - | - | - | - | - | - | - | - | - | - | - | - | + | - | + | - | + |
|  | Chronic lung disease | - | - | - | + | - | - | - | - | - | - | - | - | - | - | - | - | - | - | - |
|  | Neonatal respiratory distress | - | - | - | - | - | - | - | - | - | + | - | - | - | - | - | - | - | - | - |
|  | Breathing dysregulation | - | - | - | - | - | - | - | - | - | - | - | - | - | + | - | - | - | - | - |
| Craniofacial dysmorphisms | |  |  |  |  |  |  |  |  |  |  |  |  |  |  |  |  |  |  |  |
|  | Strabismus | - | - | - | - | + | - | - | - | - | - | - | - | - | - | - | - | - | - | - |
|  | Persistence of primary teeth | - | - | - | - | + | - | - | - | - | - | - | - | - | - | - | - | - | - | - |
|  | Pes cavus | - | - | - | - | + | - | - | - | - | - | - | - | - | - | - | - | - | - | - |
|  | External ear cartilage abnormality | - | - | - | - | + | - | - | - | - | - | - | - | - | - | - | - | - | - | - |
|  | Long fingers | - | - | - | - | - | - | + | - | - | - | - | - | - | - | - | - | - | - | - |
|  | Craniosynostosis | - | - | - | - | - | - | - | - | - | + | - | - | - | - | - | - | - | - | - |
|  | Microcephaly | - | - | + | - | - | - | - | - | - | - | - | - | - | - | - | - | - | - | - |
|  | Sacral dimple | - | - | - | - | - | - | - | - | - | + | - | - | - | - | - | - | - | - | - |
| Joint and skin | |  |  |  |  |  |  |  |  |  |  |  |  |  |  |  |  |  |  |  |
|  | Digital flexor tenosynovitis | - | - | - | + | - | - | - | - | - | - | - | - | - | - | - | - | - | - | - |
|  | Joint hypermobility | - | - | - | - | + | - | - | - | - | - | - | - | - | - | - | - | - | - | - |
|  | Patchy hypo and hyperpigmentation | - | - | - | - | + | - | - | - | - | - | - | - | - | - | - | - | - | - | - |
|  | Few cafe-au-lait spots | - | - | - | - | - | - | - | - | - | + | - | - | - | - | - | - | - | - | - |
| Feeding difficulties | |  |  |  |  |  |  |  |  |  |  |  |  |  |  |  |  |  |  |  |
|  | Oral aversion | - | - | - | + | - | - | - | - | - | - | - | - | - | - | - | - | - | - | - |
|  | Gastrostomy tube feeding in infancy | - | - | - | + | - | - | - | - | - | - | - | - | - | - | - | - | - | - | - |
|  | Dysphagia | - | - | - | - | - | - | - | - | - | + | - | - | - | - | - | - | - | - | - |
|  | Feeding difficulties | - | - | - | + | - | - | - | - | - | + | - | - | - | - | - | - | - | - | - |
| Bones | |  |  |  |  |  |  |  |  |  |  |  |  |  |  |  |  |  |  |  |
|  | Lumbar spinal canal stenosis | - | - | - | - | - | + | - | - | - | - | - | - | - | - | - | - | - | - | - |
|  | Sternum abnormality | - | - | - | - | - | - | + | - | - | - | - | - | - | - | - | - | - | - | - |
|  | Scoliosis | - | - | - | - | - | - | + | - | - | - | - | - | - | - | - | - | - | - | - |
| Others | |  |  |  |  |  |  |  |  |  |  |  |  |  |  |  |  |  |  |  |
|  | Ankyloglossia | - | - | - | + | - | - | - | - | - | - | - | - | - | - | - | - | - | - | - |
|  | Latex allergy | - | - | - | - | - | - | - | - | - | + | - | - | - | - | - | - | - | - | - |
|  | Seasonal allergy | - | - | - | - | + | - | - | - | - | - | - | - | - | - | - | - | - | - | - |
|  | Exercise induced myalgia | - | - | - | - | - | + | - | - | - | - | - | - | - | - | - | - | - | - | - |
|  | Infantile spasms | - | - | - | - | - | - | - | - | - | + | - | - | - | - | - | - | - | - | - |
|  | Single umbilical artery | - | - | - | - | - | - | - | - | - | + | - | - | - | - | - | - | - | - | - |
|  | Umbilical hernia | - | - | - | - | - | - | - | - | - | + | - | - | - | - | - | - | - | - | - |
|  | Congenital nystagmus | - | - | - | - | - | - | - | - | - | + | - | - | - | - | - | - | - | - | - |
|  | Prolonged neonatal jaundice | - | - | - | - | - | - | - | - | - | + | - | - | - | - | - | - | - | - | - |
|  | Male genitalia abnormality | - | - | - | - | - | - | - | - | - | - | - | - | - | + | - | - | - | - | - |
|  | Hypothyroidism | - | - | - | - | - | - | - | - | - | - | - | - | - | - | - | - | - | - | + |
|  | Epistaxis | - | - | - | - | - | + | - | - | - | - | - | - | - | - | - | - | - | - | - |
|  | Sinusitis | - | - | - | - | - | + | - | - | - | - | - | - | - | - | - | - | - | - | - |
| ’-', feature absent or undisclosed; '+' feature present; DD, developmental delay; ODD, oppositional defiant disorder; ADHD, attention-deficit/hyperactivity disorder; ASD, autism spectrum disorder; DMDD, disruptive mood dysregulation disorder. | | | | | | | | | | | | | | | | | | | | |
